# Therapeutic approach to fibromyalgia: a consensus statement on pharmacological and non-pharmacological treatment from the neuropathic pain special interest group of the Italian neurological society

**DOI:** 10.1007/s10072-025-08048-3

**Published:** 2025-02-21

**Authors:** G. Devigili, G. Di Stefano, V. Donadio, I. Frattale, L. Grazzi, E. Mantovani, M. Nolano, V. Provitera, S. G. Quitadamo, S. Tamburin, A. Truini, M. Valeriani, A. Furia, E. Vecchio, F. Fischetti, G. Greco, A. Telesca, M. de Tommaso, On behhalf of neuropathic pain special interest group of the Italian neurological society (sin)

**Affiliations:** 1 Fondazione IRCCS Carlo Besta, Milan, Italy; 2 Department of Human Neuroscience, Sapienza University, Rome, Italy; 3 Clinica Neurologica Bellaria Hospital, Bologna, Italy; 4 Child Neurology and Psychiatric Unit, Tor Vergata University, Rome, Italy; 5 Department of Neurosciences, Biomedicine and Movement Sciences, University of Verona, Verona, Italy; 6 Skin Biopsy Laboratory, Department of Neurology, Istituti Clinici Scientifici Maugeri IRCCS, Telese Terme, Italy; 7 Department of Neurosciences, Reproductive Sciences and Odontostomatology, University Federico II of Naples, 80100 Naples, Italy; 8 DiBrain Department, Bari Aldo Moro University, Bari, Italy

**Keywords:** Fibromyalgia, Treatments, Recommendations, Pharmacological therapy, Non-pharmacological therapy

## Abstract

**Background:**

Although fibromyalgia is a disabling disease, there is no targeted therapy for specific neurotransmitters or inflammatory mediators. Our aim was to provide neurologists with practical guidance for the management of these difficult patients based on a critical, narrative and non-systematic review of randomized controlled trials (RCTs) from the last 10 years.

**Methods:**

The members of the Special Interest Group Neuropathic Pain of the Italian Neurological Society evaluated the randomized controlled trials (RCTs) of the last 10 years and answered questions that allow a consensus on the main pharmacological and non-pharmacological approaches.

**Results:**

The neuropathic pain working group agreed on prescribing antiepileptic drugs or antidepressants in the case of comorbidities with anxiety and depression. As a second choice, experts have agreed on the association of antiepileptics and antidepressants, while they disagree with the use of opioids. Medical cannabis and nutraceuticals are promising new treatment options, although more data is needed to prove their efficacy. The neurologists agreed in suggesting physical activity at the first visit, particularly aerobic and strength training. As a second choice, they considered a cognitive behavioral therapy approach to be useful.

**Conclusions:**

Pharmacologic treatment with antiepileptic drugs and antidepressants in patients with co-occurring anxiety and depression, as well as an early nonpharmacologic approach based primarily on physical activity, may be a useful indication in contemporary neurology clinical practice. Non-pharmacological options, such as cognitive behavioral therapy and non-invasive brain stimulation NIBS, could improve evidence of efficacy and lead to relevant improvement in FM-related disability.

## Introduction

Fibromyalgia (FM) is a disabling disease characterized by chronic widespread pain, fatigue, sleep disturbances, cognitive failure, and headaches. While the diagnostic criteria are well established [1], the pathophysiology is still unknown. The central and peripheral nervous systems have been found to be affected, with concomitant central sensitization and small fiber pathology [2], justifying the clinical interest of neurologists [3]. Unfortunately, pharmacological therapies of FM are often unsuccessful, while non-pharmacological treatments are the first to be recommended [4]. The heterogeneity of the clinical pictures of FM may suggest that different pathophysiological mechanisms, including immune and inflammatory factors, genetic predisposition to small fiber impairments, and mechanisms of central sensitization phenomena, together with psychiatric comorbidities, contribute to the clinical picture of FM [5].

Currently, there is no targeted therapy for specific neurotransmitters or inflammatory mediators, and evidence is limited as there are few randomized controlled trials (RCTs) and these often include small populations and sample sizes. In addition, there is little evidence for the effectiveness of non-pharmacological treatments [6].

In this scenario, pending the best possible clarification of the understanding of the different FM phenotypes based on specific pathophysiologic features, we sought to reach a consensus among neurologists on the most appropriate treatment for FM based on a review of recent RCTs in the field of pharmacologic and non-pharmacologic treatments. Our aim was to provide clinicians with practical guidance for the treatment of these difficult patients based on a critical and narrative review of RCTs from the last 10 years.

## Methods

This was a consensus based on a narrative-systematic review of RCTs on pharmacological and non-pharmacological approaches to FM.

To this end, the active members of the Neuropathic Pain Special Interest Group of the Italian Neurological Society (NPG), who recently contributed to the diagnostic guideline for fibromyalgia [3], reviewed the most recent RCTs and answered questions that yielded a consensus on the main pharmacologic and non-pharmacologic approaches. The group of neurologists was also supported on certain arguments, such as physical activity, by external experts who were not involved in the final consensus.

The members of the NPG analyzed the RCTs of the last 10 years. For pharmacological therapies, the NPG members included antidepressants, antiepileptics and opioids and also included a chapter on cannabinoids and dietary supplements. For non-pharmacological therapies, they included acupuncture, cognitive behavioral therapy, physical activity and non-invasive brain stimulation.

NPG members searched the Pubmed database from 2014 to 2024 using specific keywords for selected pharmacological and non-pharmacological treatments (Table 1) with the following filters: Clinical trial, Phase I, II, III, Randomized controlled trial. For pharmacologic treatments, antidepressants, antiepileptics, opioids, cannabinoids, supplements, and nutrients were entered into Pubmed along with the term fibromyalgia. They selected only original studies and excluded reviews. NPG members included studies with placebos and/or comparisons with different active treatments.

**Table 1:** Search strategies for pharmacological and non-pharmacological treatments

Type of treatment	Search string	Retrieved records
Pharmacological treatments		
Antidepressants	(fibromyalgia) AND ("Antidepressive Agents" [Pharmacological Action] OR "Antidepressive Agents, Second-Generation" [Pharmacological Action] OR "Antidepressive Agents, Tricyclic" [Pharmacological Action])	40
Antiepileptic drugs	(fibromyalgia) AND ("Anticonvulsants"[Mesh] OR "Anticonvulsants" [Pharmacological Action] OR “antiepileptic drugs”)*	33
Cannabinoids	(fibromyalgia) AND (cannabis OR cannabinol OR cannabinoid* OR cannabidiol OR bhang OR hashish OR hemp OR marihuana OR marijuana OR nabilone OR cesamet OR tetrahydrocannabinol OR THC OR dronabinol OR levonantradol OR nabiximols OR palmidrol OR tetrahydrocannabinolic OR sativex OR endocannabinoid*)	10
Muscle relaxants	(fibromyalgia) AND (muscle relaxants or cyclobenzaprine or tizanidine)	0
Nutraceutics	(fibromyalgia) AND (nutraceutics)	7
Opioids	fibromyalgia) AND (opioids or tramadol or tapentadol or naltrexone or morphine or naloxone)	2
Non-pharmacological treatments		
Physical activity	(fibromyalgia) AND (exercise OR “physical activity”)	129
Acupuncture	(fibromyalgia) AND (acupuncture OR electroacupuncture OR acupuncture therapy)	23
Cognitive Behavioral Therapy	(fibromyalgia) AND (cognitive behavioral therapy OR CBT OR “cognitive behavioural therapy” OR acceptance and commitment therapy OR ACT OR mindfulness OR cognitive restructuring)	77
Non-Invasive Brain Stimulation	(fibromyalgia) AND ("non invasive brain stimulation" OR "non-invasive brain stimulation" OR NIBS OR transcranial magnetic stimulation OR TMS OR "repetitive transcranial magnetic stimulation" OR rTMS OR "theta burst stimulation" OR TBS OR transcranial direct current stimulation OR tDCS OR transcranial alternating current stimulation OR tACS OR transcranial random noise stimulation OR tRNS OR "deep transcranial magnetic stimulation" OR "deep TMS" OR neuromodulation) NOT (neurotransmitter agents OR deep brain stimulation OR DBS)	50
Diet	(fibromyalgia) AND (diet)	10

The search was run on PubMed; filters: Clinical Study, Clinical Trial, Clinical Trial, Phase II, Clinical Trial, Phase III, Clinical Trial, Phase IV, Randomized Controlled Trial, from 2014—2024

For non-pharmacological therapies, NPG members included acupuncture, cognitive behavioral therapy, physical activity, diet, and non-invasive brain stimulation (NIBS), using the same criteria as the search for pharmacological RCTs.

The main outcomes considered were pain and disability scores and the effects on other features of FM, such as sleep and fatigue.

The NPG reviewed the studies and considered those it considered most relevant. In addition to evidence of efficacy, the group also considered safety. The questions were formulated based on the reported review and a realistic consideration of the options within the Italian healthcare system and readily available services. Among the non-pharmacological approaches, the procedures that require specialized personnel and equipment but are not reimbursed within the national healthcare system were indicated as the second choice.

The neurologists then voted on a scale from 0, ‘strongly disagree’, to 10, 'strongly agree', on their level of agreement with the recommendation.

Scores from 7 to 10 were considered agreement, 5–6 for an indeterminate opinion, 0–4 for disagreement.

We applied the chi-square test to assess the distribution of opinions, and the significant prevalence of a choice was scored as definite agreement or disagreement. JMP software version 17 was used.

## Results

Table 1 contains the results of the search strategy. The following section summarises the relevant studies (Tables 1, 2 and 3).

**Table 2:** Randomized controlled trials testing the effect of pharmacological and nutraceutical treatments in fibromyalgia (FM)

Reference	Active treatment	Comparator	Sample size	Outcome measures	Findings	Adverse events (active treatment)
Antidepressants						
Upadhyaya et al., 2019 [7]	Duloxetine 30/60 mg	Placebo	184 (juvenile FM)	BPI average pain severity	Negative	Nausea 25.3%, vomiting 15.4%, headache 14.3%
Bidari et al., 2019 [8]	Duloxetine 30–60 mg	Pregabalin 75–150 mg	99	WPI, BDI-2	Positive	Nausea 34.3%, constipation 31.4%, headache 22.9%, drowsiness 20%, dry mouth 17.1%, dizziness 17.1%, insomnia: 17.1%
Pickering et al., 2018 [9]	Milnacipran 100 mg	Placebo	54	Status of CPM	Negative	Gastrointestinal disorders 28.4%, nervous system symptoms 14.7%
Ahmed et al., 2016 [10]	Milnacipran 100 mg	Placebo	19	Polysomnographic measures, BPI, FIQ	Positive for pain	Nausea/vomiting 22.2%, headache 16.7%, abdominal pain 11.1%, constipation 11.1%, sinusitis 11.8%, hot flush 11.8%
Miki et al., 2016 [11]	Mirtazapine 30 mg	Placebo	422	NRS	Positive	Somnolence 32.1%, weight gain 17.7%, increased appetite 11.6%
Murakami et al., 2015 [12]	Duloxetine 60 mg	Placebo	393	BPI, average pain score	Negative	Somnolence 26.3%, nausea 21.6%, constipation 14.9%, dizziness 5.7%, liver injury in 1 patient
Staud et al., 2015 [13]	Milnacipran 100 mg	Placebo	46	VAS, mechanical and heat pain sensitivity	Negative	Gastrointestinal disorders 10.9%
Leombruni et al., 2015 [14]	Duloxetine 60 mg	acetyl L-carnitine 1500 mg	65	VAS, MADRS, HADS-D	Positive in both arms	Nausea, anxiety, insomnia, and diarrhea in 8 patients
Combination of antidepressants + anticonvulsants						
Abdel Fattah et al., 2020 [15]	Milnacipran 100 mg + pregabalin 300 mg	Pregabalin 300 mg	58	FIQ, VAS, Leeds Sleep Evaluation Questionnaire	Negative (combination treatment not superior to pregabalin)	Disturbed sleep pattern 26.9%, dizziness and drowsiness 19.2%, gastrointestinal disorders 15.4%
Ramzy et al., 2017 [16]	Paroxetine 25 mg + pregabalin 75 mg	Pregabalin 75 mg + amitriptyline 25 mg Pregabalin 75 mg + venlafaxine 75 mg	75	SSS-8, CESDS	Positive	Dry mouth 7.7%, abnormal taste 7.7%, weight gain 11.5%
Gilron et al., 2016 [17]	Duloxetine 120 mg + pregabalin 450 mg	Placebo Pregabalin 450 mg Duloxetine 120 mg	41	NRS	Positive for active vs. placebo and pregabalin/duloxetine	Fatigue 29.4%, drowsiness 26.5%, dry mouth 23.5%, constipation 11.8%, insomnia 11.8%, headache 11.8%
Anticonvulsants (gabapentinoids)						
Karamanlioglu et al., 2021 [18]	Pregabalin + exercise	Exercise	40	pain VAS; PPT; DN4; SF36	Positive with both treatments. Pregabaline + exercise is more effective than exercise alone	Pregabalin: dizziness (82.4%), somnolence (82.4%), foot edema (17.6%), weight gain (5.9%), and constipation (5.9%)
Arnold et al., 2019 [19]	Mirogabalin (15–30 mg)	Pregabalin 300 mg Placebo	3864	ADPS; PGIC; FIQ	Negative for mirogabalin (both doses) vs placebo and pregabalin Positive for pregabalin vs placebo	no unexpected adverse events
Arnold et al., 2016 [20]	Pregabalin 75–450 mg/day	Placebo	107 (adolescents)	Pain NRS (primary); PGIC, ADPS, pain score change at week 15; 30% or 50% improvement in mean pain score; sleep quality NRS; FIQ-C	Negative for primary outcome measure, positive for secondary outcome measures	Dizziness (29.6%); Nausea (22.2%); Headache (18.5%); Weight increase (16.7%); Fatigue (14.8%)
Arnold et al., 2015 [21]	Pregabalin (300- 450 mg)	Placebo	197	Pain NRS (primary outcome measure); measures of anxiety, depression, patient function, and sleep	Positive	Dizziness (28.2%); Somnolence (19.9%); Constipation (10.5%); Nausea (9.4%)
Arnold et al., 2014 [22]	Pregabalin (165- 495 mg)	Placebo	121	LTR (primary outcome measure); pain NRS, global assessment, functional status, tiredness/fatigue, and sleep	Positive for primary endpoint	PGB (placebo) Dizziness 20.6% (20.7%) Somnolence 9.5% (10.3%) Headache 4.8% (3.4%) Fatigue 7.9% (1.7%) Nausea 7.9% (1.7%) Peripheral edema 17.5% (8.6%) Weight increase 9.5% (6.9%) Vision blurred 6.3% (0.0%) Dry mouth 4.8% (10.3%) Insomnia 11.1% (1.7%)
Nasser K et al., 2014 [23]	Pregabalin 300 mg once daily	Pregabalin 150 mg twice daily	177	Daily pain NRS (primary outcome measure). Weekly VAS for pain, fatigue, trouble sleeping, and waking unrefreshed. MSS, FIQ-R, PGIC	no significant difference between either treatment option	No differences in adverse events incidence or severity
Sawaddiruk et al., 2019 [24]	Pregabalin 150 mg + CoQ10 300 mg / day	Pregabalin 150 mg / day + placebo	11	PPT, Pain VAS, FIQ, HAMDT-A	Improvement of pain and anxiety with the association	Not reported
Cannabinoids						
van de Donk et al., 2020 [25]	Different combinations of CBD and THC (Bedrocan, Bediol, Bedrolite)	Placebo	20	Pain scores, PPT, EPT, CBD and THC plasma concentration	Bediol: 30% decrease in pain scores vs. placebo	Cough
Chaves et al., 2020 [26]	THC-rich cannabis oil	Placebo	17	FIQ	Positive	Somnolence 87.5%, dizziness 25%, mouth dryness 25%,
Opioids						
Bested et al., 2023 [27]	Low-dose naltrexone (4.5 mg)	Placebo	58	FIQR, SPIR, BPI-SF, DSIS, HADS, PD-Q, PCS, QST, pharmacokinetics	Negative	Headache, fatigue, nausea, dizziness
Nutraceutics						
Leombruni et al., 2015 [14]	ALC 1500 mg	DLX 30/60 mg	65	MADRS, HADS, VAS, DT, SF36 and CGI-I	Positive with both treatments, except for HADS anxiety subscale	Only in the DLX group (8 pts) nausea, anxiety, insomnia, diarrhoea
Salaffi et al., 2023 [28]	DLX 60 mg + PGB 150 mg + PEA 1200 mg + ALC 1000 mg	DLX 60 mg + PGB 150 mg	142	WPI, FIQR, FASmod	Positive, with greater efficacy in the add-on therapy with PEA and ALC	Not indicated
Gilron et al., 2023 [29]	ALA from 300 mg/day + PGB from75 mg/day, titrated to MTD	PGB (starting at a dose of 75 mg once daily and titrated to individual maximally tolerated dose)	54	0–10 PPI, FIQ, SF-36, MOS-SS, BPI, BDI-II, BAI, SF-MPQ. acetaminophen consumption	Absent additive benefit of combining ALA with PGB	Not frequent with combination therapy
Esposito et al., 2021 [30]	ALA 400 mg-800 mg	Placebo	210 patients, only 12 with FM	0–10 NRS and VAS, Glycemia, CRE, SGPT, SGOT	Positive for pain, no effects on laboratory tests	No AEs
Gilron et al., 2021 [31]	ALA 300 mg-1800 mg; titrated to MTD- on average 1663 mg/day	Placebo	27	0–10 NRS,, FIQ, MOS-SS, PGICS, BPI, BPI-II, SF-36, acetaminophen consumption	Negative	No statistically significant differences between placebo and ALA During titration: nausea (16%), headache (12%); at MTD: depression (4%), muscle spasms (4%). During taper/washout: headache (8%)
Schweiger et al., 2020 [32]	Migratens (ALA, CoQ10, vitamin D, Mg, TRY)	Acupunture	60	0–10 VAS, FIQ-R, FSS	Positive	Migratens: gastrointestinal side effects (15%)
Martínez-Rodríguez et al. 2020 [33]	TRY and Mg-enriched Mediterranean die	Standard Mediterranean diet	22	psychological variables Pittsburgh Sleep Quality Index 4 months	Improvement of sleep duration and efficiency in TRY Mg group, anxiety and depression	None

FIQ: Fibromyalgia Impact Questionnaire; VAS: Visual Analogue Scale; NRS: Numerical Rating Scale; BPI: Brief Pain Inventory; WPI: Widespread Pain Index; BDI-2: Beck Depression Inventory-2; CPM: conditioned pain modulation; SSS-8: Somatic Symptoms Scale-8; CESDS: Center for Epidemiological Studies Depression Scale; MARDS: Montgomery Asberg Depression Rating Scale; HADS-D: Depression subscale of the Hospital Anxiety and Depression Scale; PPT: Pressure Pain Threshold; DN4: Questionnaire Douleur neuropathique 4; SF-36: Short Form Health Survey 36; ADPS: Average Daily Pain Score; PGIC: Patient Global Impression of Change; EPT: electrical pain threshold; THC: tetrahydrocannabinol; CBD: cannabidiol; FIQR: Fibromyalgia Impact Questionnaire Revised; SPIR: Summed Pain Intensity Ratings; SOWS: Subjective Opiate Withdrawal Scale; BPI-SF: Brief Pain Inventory-Short Form; DSIS: Daily Sleep Interference Scale; HADS: Hospital Anxiety and Depression Scale; PD-Q: PainDETECT Questionnaire; PCS: Pain Catastrophizing Scale

**ALC**: Acetyl-L-carnitine; **PEA**: Palmitoylethanolamide; **DLX**: duloxetine; **PGB**: Pregabalin; **ALA**: alpha-lipoic acid; **MADRS**: Montgomery Asberg Depression Rating Scale; **HADS**: Hospital Anxiety and Depression Scale; **VAS**: Visual Analogue Scale; **DT**: Distress Thermometer; **SF36**: 36-item Short-Form Health Survey; **FASmod**: Modified Fibromyalgia Assessment Status; **PPI**: Present Pain Intensity (0–10**); FIQ**: Fibromyalgia Impact Questionnaire; **MOS-SS**: Medical Outcome Study Sleep Scale; **CGI-I**: Clinical Global Impression-Improvement; **WPI**: Wispread Pain Index; **FIQR**: revised Fibromyalgia Impact Questionnaire; **FIQ**: Fibromyalgia Impact Questionnaire; **MTD**: Maximal Tolerated Dose **BPI:** Brief Pain Inventory**; BDI-II:** Beck Depression Inventory-II**, BAI:** Beck Anxiety Inventory**; SF-MPQ:** Revised Short McGill Pain

Questionnaire Version; **AE**: advers effects; **PGIC-S**: Patients Global Impression of Change Scale; **TRY:** tryptophan; **Mg**: magnesium; **CRE**: creatinine level; **SGPT**: serum glutamic pyruvic transaminase; **SGOT**: serum glutamic-oxaloacetic transaminase; **CoQ10**: Coenzyme Q10, **DASS**: Depression Anxiety Stress Scales; **11-NRS**: 11-point pain numerical rating scale; **PSQI**: Pittsburgh Sleep Quality Index; **SF-12**: 12-item Short Form Survey; **FSS**: Fatigue Severity Scale; **PCS**: Pain Catastrophizing Scale; **EPICES**: French questionnaire Evaluation de la Précarité et des Inégalités de Santé dans les Centres d’Examens de Santé

**Table 3:** Randomized controlled trials testing the effect of non-pharmacological treatments in fibromyalgia (FM)

Reference	Active treatment	Comparator	Sample size	Outcome measures	Findings	Adverse events (active treatment)
Cognitive Behavioral Therapy						
Luciano et al., 2014 [34]	ACT	RPT, WL	156	FIQ	Positive (compared to both control arms)	-
Simister et al., 2018 [35]	ACT + TAU	TAU	66	FIQ-R	Positive	-
Laura Andres-Rodriguez et al., 2019 [36]	MBSR + TAU	TAU	70	FIQ-R	Positive	-
Perez-Aranda et al., 2019 [37]	MBSR + TAU	FibroQoL + TAU, TAU	225	FIQ-R	Positive (compared to both control arms)	-
Acupuncture						
Casanueva et al., 2014 [38]	Manual/needles as the TCM (6 sessions, 1 h/session over 6 weeks)	TAU	100	Myalgic score	Positive	Increased pain, physical discomfort
Vas et al., 2016 [39]	Manual/needles as the TCM (10 sessions, 20 min/session over 10 weeks)	Non-penetrating needles	162	VAS (pain)	Positive	Aggravation of FM symptoms (2.6%), headache (0.5%), post-acupuncture pain (1.4%)/bruising (2.6%), post-acupuncture vagal symptoms (0.7%)
Uğurlu et al., 2017 [40]	Manual/needles as the TCM (12 sessions, 30 min/session gradually decreasing over time)	Park sham device*	50	VAS (pain)	Positive (at FU)	NR
Zucker et al., 2017 [41]	Manual/needles as the TCM (8 sessions, 20 min/session gradually increasing over time)	Needles on non-traditional acupuncture points	73	VAS (pain)	Positive (only for patients with higher PPTs)	NR
Karatay et al., 2018 [42]	Manual/needles as the TCM (8 sessions, 30 min/session over 4 weeks)	Sham/simulated acupuncture^§^	75	VAS (pain)	Positive	Discomfort/bruising at the sites of needle insertion
Mist & Jones, 2018 [43]	Manual/needles as the TCM (20 sessions, 45 min/session over 10 weeks)	Education	30	FIQ-R	Positive	Bruising, transient dizziness
Garrido-Ardila et al., 2020 [44]	Manual/needles as the TCM (10 sessions, 20 min/session over 5 weeks)	FKT (active control), no trt	103	10-MWT	Negative	None
Schweiger et al., 2020 [32]	Manual/needles as the TCM (10 sessions, 30 min/session)	Nutritional supplement (Migratens® treatment)	60	VAS (pain)	Positive	None
Mawla et al., 2021 [45]	Electric acupuncture (8 sessions, 25 min/session over 4 weeks)	Mock laser acupuncture	72	BPI	Positive	NR
Physical activity						
Larsson et al., 2015 [46]	Resistance exercise program (60 min sessions over 15 weeks)	Active control group	130	Isometric knee-extension force	Positive	-
Collado-Mateo et al., 2017 [47]	Exergame (postural, coordination, aerobic, strength, mobility; 60 min sessions over 8 weeks)	Non-exercise group	83	FIQ	Positive	-
Wang et al., 2018 [48]	Four Tai Chi groups (60 min sessions over 12–24 weeks)	Aerobic group	226	FIQ-R	Positive (at FU)	-
Andrade et al., 2019 [49]	Aquatic physical training (60 min sessions over 16 weeks)	Non-exercise group	54	FIQ	Positive	-
Izquierdo-Alventosa et al., 2020 [50]	Low-intensity exercise (endurance training, coordination; 60 min sessions over 8 weeks)	Non-exercise group	32	PCS	Positive	-
Serrat et al., 2021 [51]	Multicomponent treatment (pain neuroscience education, therapeutic exercise, CBT, mindfulness + pharmacological trt; 60 min sessions over 12 weeks)	Pharmacological trt	272	FIQ-R	Positive	-
Gentile et al., 2023 [52]	Supervised home-based multicomponent PA intervention focused on aerobic and resistance training	Non-supervised aerobic exercise	34	Fibromyalgia-linked invalidity questionnaire Skin biopsy	Positive	-
Diet						
Vellisca & Latorre, 2014 [53]	Dietary elimination of MSG and aspartame	WL (no diet)	72	Pain intensity (seven-point NRS)	Negative	None
Slim et al., 2017 [54]	Gluten free diet	Hypocaloric Diet	75	Gluten sensitivity symptoms,FIQ,anxiety,depression, impression of improvement	Positive for both diets	None
Roman et al., 2018 [55]	Multispecies probiotics	Placebo	40	VAS,FIQ,anxiety, depression, cognitive evaluation	Improvement of s improved impulsivity and decision-making i	None
Mauro Martin et al., 2019 [56]	Olive tree-based supplement + IGUBAC diet	No diet	31	CGPS, PCS, FSS, FIS, anthropometric measures, blood biochemical analyses	Positive (CGPS within active arm; PCS, FSS for both arms)	None
Pagliai et al., 2020 [57]	Khorasan Wheat-Based Replacement	control wheat products	20	FIQ, WPI, SS	Positive for all scores	None
Martínez-Rodríguez et al., 2020 [33]	TRY + MG Mediterranean diet	Standard Mediterranean diet	22	PSQI, BSQ, STAI-TA, POMS-29, EAT-26	Positive (STAI, BSQ, POMS-29, EAT-26)	None
Casini et al., 2024 [58]	Personalized Mediterranean diet	Generic personalized balanced diet	84	FIQ, fatigue, anxiety, depression, ten-point NRS	Positive (FIQ, fatigue, anxiety, depression within active arm)	None
NIBS – tDCS						
Fagerlund et al., 2015 [59]	tDCS (5 sessions, 20 min/session, 2 mA on M1)	Sham	48	NRS (pain, stress)	Positive	Acute mood change (0.84%), skin redness (56.30%), sleepiness (55.46%), tingling (53.78%)
Silva et al., 2017 [60]	tDCS (20 min single session, 1 mA on DLPFC)	Sham	40	ANT	Positive	NR
Santos et al., 2018 [61]	tDCS (8 sessions, 20 min/session, DLPFC)	Sham	40	Memory (RAVLT)	Positive	NR
Brietzke et al. 2019 [62]	tDCS (60 sessions, 30 min/session, 2 mA on l-DLPFC)	Sham	20	VAS (pain)	Positive	NR
Castelo-Branco et al., 2019 [63]	tDCS (16 sessions) + aerobic exercise	Sham	148	Pain	Positive	
Matias et al., 2021 [64]	tDCS (5 sessions, 20 min/session over 5 days on M1) + functional exercise (over 8 weeks)	Sham	31	VAS (pain, anxiety), 6-MWT, BDI, FIQ	Negative	Headache, tingling, dizziness, nausea (42.85%)
Arroyo-Fernández et al., 2022 [65]	tDCS (5 sessions) + exercise	Sham + exercise, no intervention	120	Pain, FIQ, BDI	Positive	NR
Samartin et al., 2022 [66]	tDCS (15 sessions, 20 min/session on M1 or DLPFC or OIC)	Sham	130	SF-36, FIQ-R	Negative	NR
Samartin et al., 2022 [67]	tDCS (15 sessions, 20 min/session on M1 or DLPFC or OIC)	Sham	130	HADS	Positive	Tickling (42.9%), itching (46.4%), burning (28.6%)
Caumo et al., 2022 [68]	tDCS (20 sessions, 20 min/session on DLPFC)	Sham	48	PCS	Positive	NR
Loreti et al., 2023 [69]	tDCS (10 sessions, 26 min/session on M1)	Sham	35	VAS (pain)	Positive	Headache (6.47%), local tingling (5.29%), local redness (12.94%), somnolence (0.58%), local itch (2.35%), neck ache (1.76%)
Schein et al., 2023 [70]	tDCS (20 min single session, anodal l-DLPFC, cathodal r-DLPFC)	HAS	18	MEP	Positive	NR
NIBS – TMS						
Boyer et al., 2014 [71]	HF rTMS (10 sessions on l-M1)	Sham	38	FIQ	Positive	None
Fitzgibbon et al., 2018 [72]	HF rTMS (20 sessions on l-DLPFC)	Sham	26	SF-MPQ, BPI, NRS (pain), MFI-20	Positive (MFI-20)	Site discomfort (15.4%), headache (15.4%), nausea (3.8%), dizziness (3.8%), other (3.8%)
Altas et al., 2019 [73]	HF rTMS (15 sessions on l-M1 or l-DLPFC)	Sham	30	VAS, FIQ, FSS, SF-36, BDI	Positive	NR
Cheng et al., 2019 [74]	HF rTMS (10 sessions on l-DLPFC)	Sham	20	VAS (pain)	Positive	None
Tanwar et al., 2020 [75]	LF rTMS (20 sessions on r-DLPFC)	Sham	90	NRS (pain)	Positive	Headache (2%)
Bilir et al., 2021 [76]	HF rTMS (10 sessions on l-DLPFC)	Sham	20	VAS, FSS, HADS	Negative	None
Izquierdo- Alventosa et al., 2021 [77]	HF rTMS (10 sessions on l-DLPFC)	Sham, physical exercise	49	VAS (pain)	Positive	NR
Lacroix et al., 2021 [78]	HF rTMS (15 sessions on l-M1)	Sham	78	VAS, PGIC	Positive	None
Argaman et al., 2022 [79]	HF rTMS (10 sessions on M1)	Sham	27	BPI, MPQ, FIQ, SF-36, STAI, BDI	Positive	NR
Pareja et al., 2022 [80]	rTMS (8 sessions) + pharmacological trt	Pharmacological trt	560	FIQ, WPI, SSS	Positive	NR
NIBS – combined						
Forogh et al., 2021 [77]	rTMS or tDCS (3 sessions, 20 min/session of rTMS or tDCS on DLPFC)	None	30	VAS (pain), FIQ-R, DASS-21	Positive (rTMS, VAS)	Mild, transient headache (rTMS)

ACT: Acceptance and Commitment Therapy; ANT: Attention Network Test; BDI: Beck Depression Inventory; BPI: Brief Pain Inventory; BSQ: Body Shape Questionnaire; C2: occipital nerve area; CGPS: Chronic Pain Grade Scale; ctrl: control; DASS-21: Depression Anxiety Stress Scale-21 Item; DLPFC: dorsolateral prefrontal cortex; EAT-26: Eating Attitudes Test-26; FibroQoL: multicomponent intervention for fibromyalgia; FIQ(-R): Fibromyalgia Impact Questionnaire (- Revised); FIS: Fatigue Impact Scale; FSS: Fatigue Severity Scale; FU: follow-up; HADS: Hospital Anxiety and Depression Scale; HAS: hypnotic analgesia session; IGUBAC: Inflammatory Gut-Brain Axis Control Diet; HF: high frequency; l: left; MBSR: Mindfulness-Based Stress Reduction; LF: low frequency; MEP: motor evoked potential; MFI-20: Multidimensional Fatigue Inventory; MFIS: Modified Fatigue Impact Scale; MG: magnesium-enriched; MSG: monosodium glutamate; M1: primary motor cortex; NIBS: non-invasive brain stimulation; NR: not reported; NRS: Numerical Rating Scale; OIC: operculo-insular cortex; PCS: Pain Catastrophizing Scale; PGIC: Patient Global Impression of Change; POMS-29: Profile of Mood States; PPTs: pain pressure thresholds; PSQI: Pittsburgh Sleep Quality Index; RAVLT: Rey Auditory Verbal Learning Test; r: right; RPT: recommended pharmacological treatment; rTMS: repetitive transcranial magnetic stimulation; SDBOLD: blood-oxygenation-level-dependent signal variability detected with functional magnetic resonance imaging; SF-MPQ: Short-Form McGill Pain Questionnaire; SF-36: Short Form 36 for health status survey; STAI-TA: State Trait Anxiety Inventory – trait anxiety; TAU: treatment as usual; TCM: traditional Chinese medicine; tDCS: transcranial direct current stimulation; 10-MWT: 10 m walking test; trt: treatment; TRY: tryptophan; VAS: Visual Analogue Scale; WL: waiting list

^*^Non-penetrating needle device with a blunt and retractable needle and a guide tube

^§^Needles inserted into points that are not recognized as acupoints or in meridians /simulated: needles into the bandage but not into the skin

### Pharmacological therapy

Antidepressants. From the 40 clinical trials reviewed, we selected eleven randomised controlled trials (RCTs) that investigated the effect of antidepressants in patients with fibromyalgia (Table 2). Two placebo-controlled trials, involving a total of 577 patients, investigated the effect of duloxetine. [7, 12]. Although the primary endpoint was not significantly improved by duloxetine compared to placebo, treatment with duloxetine was associated with an improvement in secondary endpoints, including analgesia and quality of life. An RCT comparing the effects of duloxetine and pregabalin showed an improvement in WPI scores with a statistically significant difference between the two treatment arms in favour of duloxetine [8]. An RCT compared the effect of duloxetine and acetyl-L-carnitine on pain, depression and anxiety [14]. Both drugs led to an overall clinical improvement, with positive effects on pain and depressive symptoms. Three RCTs with a total of 119 patients investigated the effect of milnacipran 100 mg [9, 10, 13]. Treatment with milnacipran showed no significant reactivation of conditioned pain modulatory function [9] and was not superior to placebo in reducing clinical pain and mechanical and thermal pain sensitivity in quantitative sensory tests [13]. Ahmed and colleagues investigated the effect of milnacipran on polysomnographic measures of sleep and pain. This drug did not significantly improve the polysomnographic parameters, but showed a significant improvement in daily pain perception and subjective sleep quality [10]. In an RCT involving 430 patients, the effect of mirtazapine on FMG was investigated [11]. The authors found a greater reduction in the mean NRS pain score from baseline to the end of treatment with mirtazapine (30 mg) compared to placebo [11]. Three RCTs investigated the efficacy of combination treatment with antidepressants and pregabalin [15–17]. The combination of pregabalin (450 mg) and duloxetine (120 mg) improved several clinical outcomes compared to pregabalin monotherapy [17]. Pregabalin (300 mg) in combination with milnacipran (100 mg) showed adequate efficacy in the treatment of patients with FMG, but no superiority over monotherapy [15]. The combined use of pregabalin (75 mg) plus paroxetine (25 mg) improved somatic and depressive symptoms compared to pregabalin plus amitriptyline (25 mg) or venlafaxine (75 mg) [16].

Recent studies have investigated the effect of dietary supplements as add-on therapy to gabapentinoids and antidepressants, as explained in the following paragraph (Table 2).

Although more RCTs are needed to draw convincing conclusions about efficacy in FM, antidepressants in monotherapy or in combination with a2d ligands are considered a valid treatment option for patients with FM. Minor side effects are common, but serious events are rare.

Antiepileptic drugs (gabapentinoids). According to the search strategy of this review, we screened 33 clinical trials on anticonvulsants in fibromyalgia and selected ten randomized controlled trials with a total of 4715 patients (Table 2).

Most studies investigated the efficacy of pregabalin alone or in combination with other medications compared to placebo. In particular, two RCTs showed the efficacy of PGB compared to placebo on pain and related symptoms in adults [21, 22] and one study in adolescents with FM [20] failed to show a significant improvement in mean pain score. The association with CoQ10 showed an improvement in the efficacy of PGB [24]. The efficacy of PGB at a dose of 150 mg twice daily was found to be no better than a single daily dose of 300 mg [23]. The combination of PGB with exercise was more effective than exercise alone [18]. The combination with duloxetine showed greater efficacy than placebo and than the two monotherapies [17], while a study directly comparing the efficacy of PGB with that of duloxetine showed the superiority of duloxetine [8]. The combination with another antidepressant such as milnacipran did not show superiority compared to treatment with PGB alone [15]. Mirogabalin, a gabapentinoid that binds to the α2δ-subunit of the voltage-gated calcium channel and has significantly higher efficacy, was found to be safe but less effective than PGB and placebo at a dose of 15 mg once or twice daily [19]. Although gabapentinoids are generally effective, their use often causes side effects such as dizziness, drowsiness, headache and fatigue.

Medicinal cannabis there are numerous clinical studies on cannabis and D-9-tetrahydrocannabinol (THC) for chronic pain conditions. However, there is clinical evidence for the use of CBD in FM showing a wide-ranging therapeutic effect [81]. Unfortunately, there is only weak evidence of efficacy as CBD + THC therapy has not been thoroughly tested in large clinical trials.

A Cochrane review published in 2016 examined 2 randomized controlled trials of cannabinoids in FM involving 72 patients. Both studies investigated the effect of nabilone, a derivative of Delta 9-THC. In the study by Shrabek et al. nabilone was compared with placebo, in the other study with amitriptilyne. The authors described good results in terms of pain and sleep, but with a low level of evidence, short-term treatment and insufficient data on important outcome measures such as disability scores and psychopathological variables [25].

Two RCTs have been published in the last 10 years. Van de Donk et al. [25] tested 4 different cannabis strains: Bedrocan (22.4 mg THC, 0.1 mg CBD); Bediol (13.4 mg THC, 17.8 mg CBD); Bedrolite (18.4 mg CBD, 0.1 mg THC); and a placebo strain without THC or CBD, by inhalation). They evaluated the relief of experimental pressure pain, electrical pain and spontaneous pain (primary endpoints) as well as the subjective and psychotropic effects. The analgesic efficacy of the active treatment was limited, but strains containing THC plus CBD-bediol showed a 30% effect on all pain scores. The study by Chaves et al. [26, 82] aimed to determine the benefits of a tetrahydrocannabinol (THC)-rich cannabis oil on the symptoms and quality of life of fibromyalgia patients. They used a THC-rich cannabis oil (24.44 mg/mL THC and 0.51 mg/mL9) compared to placebo in 17 women with fibromyalgia and observed a significant improvement in FIQ scores in the treated group.

In summary, there are few RCTs with weak evidence for the efficacy of CBD and THC in the treatment of FM, raising doubts about the best therapeutic formulation, route of administration and timing of treatment (Table 2). However, numerous observational studies appear to indicate good tolerability and therapeutic efficacy of medical cannabis in patients with FM [83], with a focus on cannabidiol [84, 85]. Given the paucity of pharmacological options for FM, an RCT on the combination of THC and cannabidiol (bediol) could be of great benefit.

Opioids—There are few randomized controlled trials that have investigated the efficacy of opioids in fibromyalgia. Most of these studies were published more than 10 years ago.

More than 10 years ago, Sorensen et al. [82, 84] reported on two different studies investigating the effect of morphine (0.3 mg/kg) on pain relief using different clinical scales. The conclusion from these studies was that morphine did not significantly improve pain, but few side effects such as nausea and/or vomiting were reported by patients. Later, tramadol (100 mg) was tested in fibromyalgia in a randomized controlled trial [85] in a small group of patients. This study found that tramadol led to a reduction in spontaneous pain, while no difference was found in objectively measured pain (pressure dolorimetry). A more reliable study was conducted by Russel et al. [86], which investigated tramadol (50–400 mg) in a large cohort of patients. The results of this study support the efficacy of tramadol over a 6-week period for the treatment of fibromyalgia pain, assessing the cumulative probability of discontinuation of the double-blind study due to inadequate pain relief. The combination of tramadol (37.5 mg) with paracetamol (325 mg) was then tested [87]. The main outcomes assessed were cumulative time to treatment discontinuation, pain relief, total number of tender points and specific questionnaires*.* The conclusion of this study was that this combination is effective in the treatment of fibromyalgia pain and has no serious adverse effects. Younger et al. [88] used naltrexone (50 mg/die) in a small group of fibromyalgia patients. Outcomes studied included changes in sensitivity to heat, cold, and mechanical pain, as well as measures of mood and opioid withdrawal symptoms. The authors concluded that naltrexone had no effect on pain and mood in fibromyalgia patients, suggesting that endogenous opioid activity is not involved in the pathophysiology of fibromyalgia. In addition, the effect of low-dose naltrexone (4.5 mg/day) on daily self-reported pain and overall life satisfaction, positive mood, sleep quality and fatigue was investigated in a small cohort of patients [89]. This study showed that low-dose naltrexone has a clinically beneficial effect on fibromyalgia pain without serious side effects. However, the conclusion of this study was not confirmed in a trial of low-dose naltrexone involving a larger cohort of fibromyalgia patients [27]. In fact, no clinically relevant analgesic efficacy of low-dose naltrexone treatment was observed in these patients when using the revised Fibromyalgia Impact Questionnaire scores and the sum of pain intensity ratings as primary outcomes.

To summarize, there are only a few RCTs, mostly from the last 10 years, investigating the efficacy of opioids and their antagonists. However, with the exception of tramadol, there is no clear evidence of the efficacy of these drugs for pain relief in fibromyalgia patients. Of the drugs studied, morphine and naltrexone showed no clear effect, while tramadol showed positive effects on pain relief, although objective evidence of its efficacy was lacking.

Given these contrasting results, further RCTs on these drugs, particularly tramadol, are desirable to establish objective measures.

Muscle relaxant drugs. In the last 10 years, research into RCTs to demonstrate the effect of muscle relaxant drugs has not yielded results. In a study conducted more than 10 years ago, Reynolds et al. conducted a double-blind cross-over study of cyclobenzaprine at an evening dose of 10 or 30 mg and found a positive effect on tender points, anxiety, stiffness, and fatigue at the 10-mg dose, with fewer side effects than at the other dose [90]. Cyclobenzapine is currently used in Italy and is also considered as therapeutic support in other recommendations [4], but the lack of recent studies does not allow to formulate an opinion on its possible efficacy.

Nutraceutical products. The effect of dietary supplements in fibromyalgia has recently been investigated in a few studies, but these had methodological limitations, such as small sample sizes and, in many cases, the concomitant use of other therapeutic strategies [28, 29, 31, 32, 89, 91]. Acetyl-L-carnitine (ALC) has only been tested in one study of FM [92], but its usefulness in relieving neuropathic pain [93] and improving depressive symptoms [94, 95] provides a rationale for its use. A recent RCT studied 65 female FM patients randomised to receive either duloxetine 60 mg/day or ALC 1500 mg/day p.o. and found a significant improvement in mood/depressive state, severity of illness and physical well-being. However, there was no significant reduction in VAS pain scores, and neither drug improved anxiety.

Palmitoylethanolamide (PEA) is an endogenous lipid mediator with neuroprotective, anti-inflammatory and analgesic effects. To date, only three studies have investigated the effect of PEA in fibromyalgia. Two of these were observational studies that showed a positive effect on pain and limited adverse effects [89, 91]. Only one RCT aimed to evaluate the effects of PEA (600 mg b.i.d.) in combination with acetyl-carnitine in FM patients already receiving duloxetine and pregabalin*.*The addition of these supplements to conventional pharmacological therapies led to significant improvements in all outcomes assessed (WPI, FIQR, FASmod) [28].

Alpha-lipoic acid (ALA) is an antioxidant and immunomodulatory agent that plays an important role in various metabolic processes. In the CADENCE study, an ALA-pregabalin combination (with a randomized, double-blind, 3-phase crossover design) was investigated (over 6 weeks). This study provided no evidence of additional benefit of ALA compared to pregabalin in terms of daily pain and all secondary endpoints. The maximum tolerated doses of ALA and pregabalin were similar for both combination therapy and monotherapy, and adverse events (AEs) were rare [29].

The IMPALA study (a double-blind, randomized, placebo-controlled crossover study with a small sample size over 10 weeks) showed no beneficial effects of ALA compared to placebo, with no significant adverse events (good tolerability) [31]. Contrasting results (to the IMPALA study) were obtained by Esposito et al. [30] in a (monocentric, randomized, double-blind, placebo-controlled) clinical trial involving only 12 patients with fibromyalgia (who needed an alternative treatment to conventional painkillers because they were unable or unwilling to take painkillers). They reported benefits (with ALA 400–800 mg/day) in terms of pain intensity with no adverse effects (in particular, no hepatic toxicity and no effect on blood glucose levels).

A randomized, controlled study compared the effect of a nutrient combination containing coenzyme Q10, vitamin D, ALA, magnesium and tryptophan (Migratens) with the effect of acupuncture over a period of 3 months. This showed a reduction in pain and an improvement in quality of life (FIQ-R and FSS) with more limited effects for Migratens compared to acupuncture, despite a higher number of discontinuations due to side effects [32]. These authors recommend cyclical use to improve long-term compliance.

Magnesium (MG) supplementation has been suggested to alleviate the various symptoms associated with fibromyalgia, reduce certain types of pain and improve the central nervous system's ability to withstand stress. In recent years, only one RCT examined the effects of MG on stress in 76 patients with fibromyalgia, including pain, sleep, quality of life, fatigue, catastrophizing, social vulnerability, and MGg blood concentrations [33, 34, 96]. The results show that magnesium improves mild/moderate, but not severe, stress and reduces pain perception in fibromyalgia patients.

Ultimately, the use of dietary supplements containing ALC, PEA and MG is promising for the treatment of FM in conjunction with pharmacologic therapies, but the data are inconclusive due to the lack of clinical trials with sufficient sample size on larger series.

### Non pharmacological approach

Behavioral approach—Cognitive Behavioural Therapy (CBT) CBT enables patients to change their negative thoughts, feelings and expectations. This can improve mood, improve the ability to cope with pain and change the perception of pain. The pharmacological option can be complemented by CBT so that patients can become more aware of their condition.

Recent clinical experience confirms the benefits of CBT in the treatment of fibromyalgia [97, 98]. A review published in 2021 by Heller [99] evaluated the latest literature and confirmed that CBT in combination with pharmacological treatment can help patients with fibromyalgia to improve pain and depression as well as catastrophizing attitudes. Some reports have shown an effect of CBT on brain connectivity [100, 101].

Unfortunately, the clinical experience, although encouraging, is based on results obtained from small patient groups, short follow-up periods and studies without control conditions, so the results cannot be considered conclusive. The complexity of the clinical condition is considerable and it is likely that a single pharmacological option will not be sufficient: it is imperative that we develop multidisciplinary treatment programmes, including behavioural approaches such as CBT, to help patients with fibromyalgia.

Acceptance Commitment Therapy (ACT)—ACT belongs to the third wave of behavioral therapy approaches. It aims to improve patients' functioning and quality of life by increasing psychological flexibility, defined as the ability to observe and accept aversive and disturbing thoughts, emotions and bodily sensations without reacting to them, and to promote behavior consistent with personal values [96].

The EFFIGACT study, a 6-month randomized controlled trial that enrolled and randomized 156 patients, showed that ACT was statistically superior to both the recommended pharmacological treatment (RPT: pregabalin + duloxetine) and the waiting list immediately after treatment, and improvements were maintained after 6 months [36, 37]. It also showed that ACT appears to be a cost-effective treatment compared to RPT, as the ACT group incurred fewer direct costs compared to the two control arms during the 6-month study period due to lower costs for primary care visits and FM-related medications [34].

In addition, Varallo et al. have shown that acceptance interventions are effective in reducing kinesiophobia and improving performance-related physical functioning in individuals with comorbid obesity, as they can facilitate adherence to physical activity and promote weight loss in this highly comorbid clinical condition [102].

In addition, there is preliminary evidence that online delivery of ACT therapy is an effective, accessible and cost-effective treatment for people with FM and other chronic pain conditions [35].

Mindfulness-Based-Stress-Reduction (MBSR)—Mindfulness has gained attention among third-wave behavioral therapy interventions in the last year, and there are studies demonstrating the use of mindfulness for various forms of pain.

The Mindfulness-Based Stress Reduction (MBSR) program is a multi-component intervention that aims to alleviate suffering through the cultivation of mindfulness: Mindfulness is an attitude of “awareness that arises through conscious attention in the present moment and nonjudgmental observation of the unfolding experience” [100]: It is a helpful complementary treatment for patients with fibromyalgia, as it improves some of the main symptoms of fibromyalgia and reduces the subjective burden of the disease [101].

The EUDAIMON study, a 12-month randomized controlled trial, included 225 participants with fibromyalgia who were randomly assigned to 3 study arms: MBSR plus usual care (TAU), FibroQoL (multicomponent intervention for FM) plus TAU, and TAU alone. The results showed that functional outcomes and symptoms in fibromyalgia were moderately improved by MBSR plus usual care compared to usual care alone [37].

Other references confirm the benefits of MBSR in FM as an effective intervention to reduce the clinical severity of patients with FM. In addition, MBSR can modulate immune-inflammatory signaling pathways relevant to the pathophysiology of FM [36] (Table 3).

In summary, although the data are preliminary and based on studies without control conditions or long-term follow-up, it is reasonable to consider behavioural approaches to support patients suffering from FM during their difficult therapeutic strategies to provide them with tools that enable them to endure and manage pain and improve their quality of life and disability resulting from the disease.

Acupuncture. From the 23 screened clinical trials, nine randomized controlled trials (RCT) were selected to investigate the effect of acupuncture in patients with fibromyalgia (Table 3), involving 725 patients. Eight studies used the Traditional Chinese Medicine [TCM] needle acupuncture technique, while one study used electroacupuncture (EA) (i.e. AS Super 4 Digital Needle Stimulator) with low-frequency stimulation [45]. The predominant sham procedure was a non-penetrating needle procedure using a blunt and retractable needle and guide tube or placement on non-traditional acupuncture points [39, 40], and better efficacy of true acupuncture compared to sham acupuncture was found. Interestingly, Uğurlu et al. [40, 46] and Karatay et al. [42] described a significant improvement in pain intensity, functional impairment and emotional distress 2 and 3 months after treatment, respectively, although a consistent placebo effect after sham acupuncture was found only in the short term (1 month). Three studies did not use sham stimulation but alternative control conditions, namely a group discussion of a book on the current understanding of fibromyalgia [43], a dietary supplement [32], physiotherapy [44] or treatment as usual [38]. In three of these studies, the authors found clinically and statistically significant improvement in the acupuncture group compared to the control groups, not only in terms of pain intensity and functional outcome [43, 44] but also in terms of other secondary measures such as pressure pain thresholds, emotional distress and fatigue, activity engagement, general health status and global subjective improvement [38], at post-treatment and follow-up assessments. Only in the RCT comparing acupuncture with physiotherapy was true acupuncture found to be as effective as the control treatment [44]. A notable result was found in the study by Zucker and colleagues [41], who investigated the effects of needle acupuncture taking into account patients’ baseline pressure pain threshold (PPT). The authors found that patients with a higher pressure pain threshold experienced more significant pain relief after active acupuncture. In contrast, participants with lower PPTs showed a stronger pain-relieving response to sham acupuncture [41]. Finally, only one selected RCT [45] examined the neural correlates of electrical acupuncture stimulation. Consistent with previous studies, the results reported that active acupuncture treatment reduced pain intensity more compared with sham treatment, although there was no difference in terms of acupuncture sensations and treatment credibility.

In most studies, points were standardized among participants [38, 40, 42–45] considering FM tender points of fibromyalgia defined according to the 1990 ACR classification criteria [103] or predominant FM symptoms [38, 41, 42, 45]. In the remaining three studies, the stimulation points were subjectively selected by experienced acupuncturists based on the individual patient [32, 39, 43]. The average duration of each stimulation was about 30 min and was repeated on average twice a week or once a week and then gradually increased/decreased to several times a week by the end of the treatment period. The longest treatment duration of the included studies was 10 weeks. Only two studies did not investigate follow-up measures. All but one study [44] reported long-term efficacy of acupuncture stimulation, and the longest follow-up period was 1 year [39] (Table 3). Further RCTs are needed to clearly demonstrate the efficacy of acupuncture for fibromyalgia. Mild side effects are frequently observed, but serious adverse events are rare. There are some differences between the various procedures in terms of acupuncture points, duration and number of stimulations.

Physical activity Exercise guidelines for fibromyalgia patients are based on a limited literature similar to that for healthy adults, but can be supplemented by recommendations from seven RCTs that examined various exercise interventions with 831 patients (Table 3). A manual selection of the most relevant RCTs was made based on sample size, methodological/statistical rigor applied, and the types of exercise protocols specified in the main generally accepted guidelines.

Larsson et al. [46] investigated resistance training and found an improvement in muscle strength and pain intensity. Collado-Mateo et al. [47] studied exergames and reported improvements in quality of life and pain. Wang et al. [48] compared Tai Chi with aerobic exercise and found similar improvements in fibromyalgia symptoms. Andrade et al. [49] investigated the effects of aquatic exercise and found a possible benefit in alleviating symptoms. Izquierdo-Alventosa et al. [50] studied low-intensity physical exercise and found a reduction in pain catastrophizing and improvements in psychological and physical aspects. Serrat et al. [51] investigated a multicomponent treatment that was shown to be effective in symptom control through neuroscience pain education, therapeutic exercise, cognitive behavioural therapy, and mindfulness. Finally, Gentile et al. [52] found that physical activity at home improved small fibre pathology and disease severity in a group of fibromyalgia patients (Table 3).

In accordance with the guidelines for healthy adults, some considerations are fundamental:

- Increasing the frequency of physical activity (PA) to 3 days per week is associated with a greater reduction in symptoms than 1–2 days per week.
- It is important to ensure adequate rest between exercises by alternating different body parts to optimize effectiveness.
- If an uninterrupted 30-min aerobic exercise session is not tolerated initially, breaking it up into shorter sessions and providing more support may improve adherence to the program [46, 104].

The rate of progression within the FITT-VP (Frequency, Intensity, Time, Type-Volume Progression) framework depends on symptoms, so it is necessary to adjust the intensity or duration of PA during a flare-up. Minimizing the eccentric components of resistance exercise reduces muscle microtrauma during flare-ups [46, 50, 104].

Individuals suffering from fibromyalgia fall into physical inactivity due to symptoms. The prescription of PA should be based on pain tolerance and should be gradual while pain levels are monitored [50, 104]. Structuring exercise programs to minimize barriers and accommodate individual preferences can improve adherence. This is best done in supervised or group sessions that encourage social support [51].

Functional activities with gradually increasing intensity, from light to moderate, are recommended despite the symptoms [50]. Proper demonstration of exercise biomechanics limits the risk of injury and PA in a controlled environment (temperature and humidity) can mitigate symptom exacerbation [46, 104].

Both land-based and aquatic aerobic PA, together with exergames, are useful to improve physical function and reduce pain [47, 49, 104]. Complementary therapies such as Tai Chi and yoga also alleviate symptoms [48].

Overall, these studies highlight the importance of non-pharmacological interventions and in particular aerobic exercise and resistance training, possibly personalized and supervised, in the treatment of fibromyalgia and provide insights into the association with different approaches to alleviate symptoms and improve quality of life. However, further research is needed to investigate the long-term effects and optimal combinations of these interventions.

Diet—Among the causes unrelated to the specific causes of central sensitization, a fundamental mechanism in FM, is inflammation [105]. Inflammation consists of a dynamic sequence of phenomena manifested in an intense vascular response and, above all, in the release of endogenous substances: the chemical mediators of inflammation [106].

Diet can cause inflammation. Inflammatory conditions of the digestive system can trigger the release of cytokines, which can have effects on the central nervous system [107].

There is a general impression that a plant-based, Mediterranean, vegetarian and vegan diet can reduce musculoskeletal pain, especially in patients with rheumatoid arthritis [108].

However, there is only weak evidence for the beneficial effect of specific diets in FM, although studies suggest that weight control, a modified diet high in antioxidants and supplementation are beneficial in relieving symptoms [109].

Among the RCTs addressing the effects of diet on the symptoms of FM, seven described the effects of different dietary approaches on FM symptoms.

Mauro-Martín et al. [56] analyzed the effects of an olive tree-based supplement and a gluten-free, and low histamine diet (IGUBAC-Diet®), with antioxidant and anti-inflammatory characteristics, in women with fibromyalgia in a RCT trial with 31 women with FM They found a general improvement in pain, fatigue and quality of life.

Another RCT investigated the effects of avoiding monosodium glutamate (MSG) and aspartame on pain perception in fibromyalgia. However, eliminating MSG and aspartame from the diet did not lead to an improvement in fibromyalgia symptoms [53].

A recent RCT investigated the effects of a Mediterranean diet enriched with tryptophan (TRY) and magnesium (MG) on psychological variables (anxiety, mood, eating disorders, self-image) and sleep quality in women with fibromyalgia [33].

The intervention group received a Mediterranean diet enriched with high doses of TRY and MG (60 mg TRY and 60 mg MG), while the control group received the standard Mediterranean diet.

Patients receiving the supplement showed improvements in anxiety, depression and eating disorders. [33] The khorasan wheat replacement diet led to an improvement in key FMS indices such as WPI, SS and FIQ compared to the control wheat diet. The clinical improvement corresponded with the changes in the gut microbiota [57]. Roman et al. investigated the effect of supplementation with probiotics of different species on clinical symptoms and cognitive profile in patients with FM and found a positive effect on impulsivity and decision making [55, 70, 110]. Another study investigated the differential effects of a gluten-free diet compared to a hypocaloric diet in 75 patients with cystic fibrosis randomly assigned to the two diets. Both diets had positive effects on clinical symptoms and gluten sensitivity [54].

A recent RCT [58] studied 84 cystic fibrosis patients, 39 of whom followed a personalized Mediterranean diet (DIET group) and 45 a general balanced diet (NODIET group). Patients in the DIET group showed improvement in most fibromyalgia parameters, including disability score, fatigue and anxiety (Table 3).

In conclusion, further studies are needed to determine the best nutritional support for FM.

A hypocaloric Mediterranean-type diet with probiotics and magnesium and tryptophan supplementation appears to have a beneficial effect on FM.

Non invasive brain stimulation (NIBS) The therapeutic options for fibromyalgia (FM) are limited, and medications, which are often associated with side effects, play only a marginal supportive role [77]. Non-invasive brain stimulation (NIBS) techniques such as transcranial direct current stimulation (tDCS) and repetitive transcranial magnetic stimulation (rTMS) are safe and well-tolerated options used for many pain conditions, including FM [111, 112]. Here we summarize the most important randomized controlled trials (RCTs) investigating the efficacy of NIBS in the treatment of FM.

Transcranial direct current stimulation—tDCS. TDCS is a neuromodulation technique that can increase the excitability of corticospinal pathways [70]. It uses direct current applied to the scalp via electrodes to induce changes in cortical activity and excitability [110, 113]. In FM patients, stimulation of target areas such as the primary motor cortex (M1) [59], dorsolateral prefrontal cortex (DLPFC) [62, 68] and operculo insular cortex (OIC) [66, 67] improves fatigue [69], quality of life (QoL) [69], pain catastrophizing and pain-related disability [68]. Transcranial direct current stimulation –tDCS- via the DLPFC improves specific cognitive functions related to episodic short- and long-term memory and executive functions [61], while no effects on alertness were found [60].Improvements in pain perception [62] have been demonstrated with lower visual analogue scale (VAS) scores and depressive symptoms [67] as assessed by the Fibromyalgia Impact Questionnaire (FIQ) and Beck's Depression Inventory (BDI) [65].

Conflicting data are available on tDCS in combination with physical exercise [63–65].

Repetitive transcranial magnetic stimulation – rTMS. The effects of rTMS are mediated by an electromagnetic field over the scalp, which modulates the excitability of both deep brain and cortical areas [114]. In FM patients, rTMS of the DLPFC improves fatigue and pain [72, 74, 75]. When considering physical functioning, depression and perception of general health status as outcomes, rTMS of the left premotor cortex (PMC) is more effective than rTMS of the left DLPFC [73]. It should be mentioned that the effect of rTMS of the left DLPFC compared to sham stimulation on pain, stiffness, fatigue, quality of life, mood and cognitive state is controversial [76].

The rTMS-induced improvements in quality of life occur via the metabolism of the right limbic system, which may be the neural substrate of the rTMS effect on emotional dimensions [71]. It is noteworthy that studies show that the improvement in FM symptoms induced by rTMS lasts up to 6 months after the last stimulation [78, 80, 115]. Other studies [77, 79] compared the effects of rTMS and tDCS on pain and quality of life in FM patients and showed a higher and longer lasting analgesic effect of rTMS (Table 3).

In conclusion, although more data are needed to support the benefits of NIBS in fibromyalgia, the available studies are encouraging and show that both tDCS and rTMS not only lead to pain reduction but could also have a positive effect on the complex galaxy of symptoms typical of the disease.

## Consensus

After the presentation of the RCT revisions, the members of the NPG (N° 13) answered the following questions.

1. Patients with fibromyalgia should take pharmacological treatment after the initial diagnosis
2. Antidepressants should be used as a first approach (duloxetine 30/60 mg, * mirtazapine 30 mg/day)
3. Antiepileptic drugs should be used as a first approach (pregabalin 150—300 mg/day) *
4. The choice between antidepressants or antiepileptics depends on clinical assessment (comorbidity with depression and/or anxiety)
5. Integrators and nutrients should be used as first choice
6. Opioids should be suggested on first approach
7. The combination of antiepileptics and antidepressants should be suggested in patients who do not respond to monotherapy
8. Cannabis should be used in drug-resistant patients
9. The following treatments would be worth further evidence: medical cannabis
10. The following treatments would be worth further evidence: opioids (tramadol, tapentadol)
11. The following treatments would be worth further investigation: nutraceutical products
12. Physical activity (supervised multicomponent activity including aerobic and resistance training) should be suggested in the first approach
13. A personalized Mediterranean diet should be prescribed at the first approach
14. Cognitive behavioral therapy (acceptance and commitment and/or mindfulness) should be prescribed as a second-line non-pharmacological approach
15. Motor or dorsolateral prefrontal cortex TMS or TDCS should be suggested as a * second-line non-pharmacological approach
16. Traditional Chinese medicine acupuncture should be used as a second non-pharmacological option

**Fig. 1:**
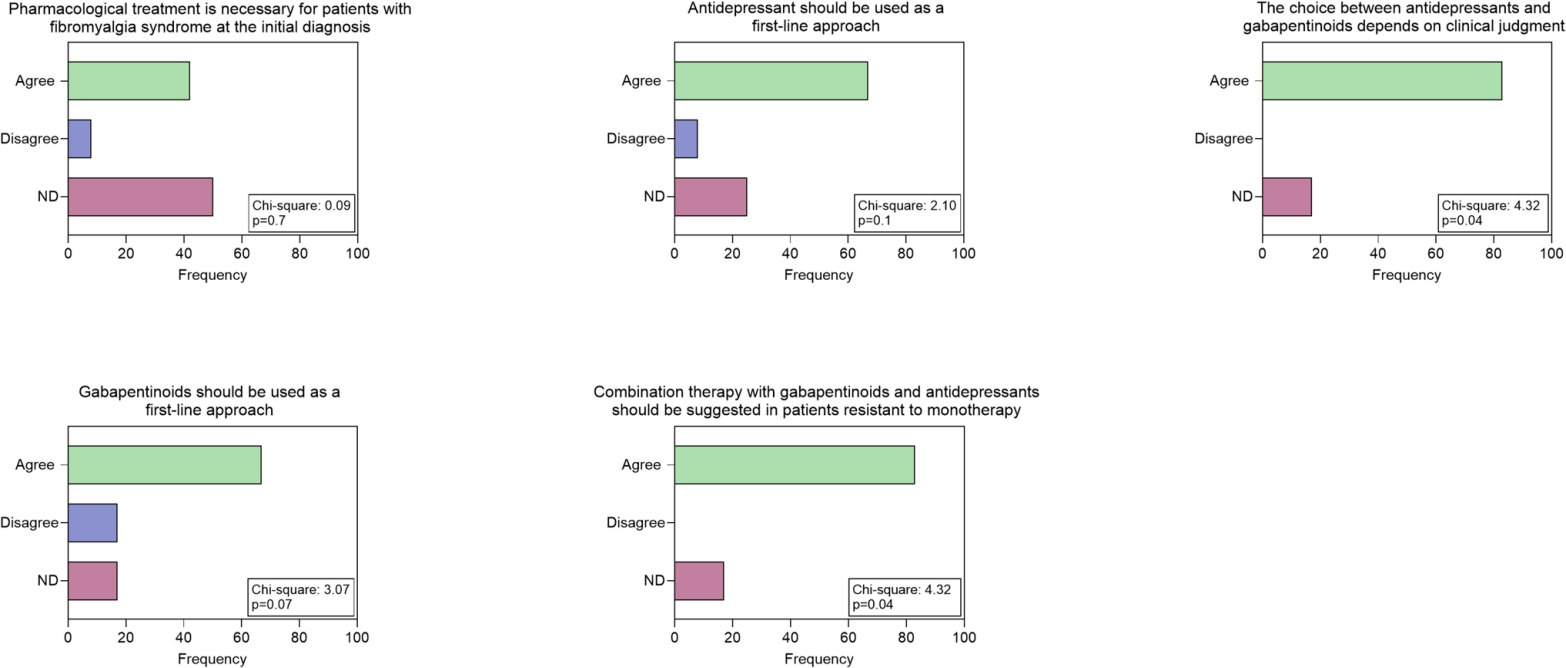
Answers to questions about pharmacological therapies: Chi-square test results are indicated

**Fig. 2:**
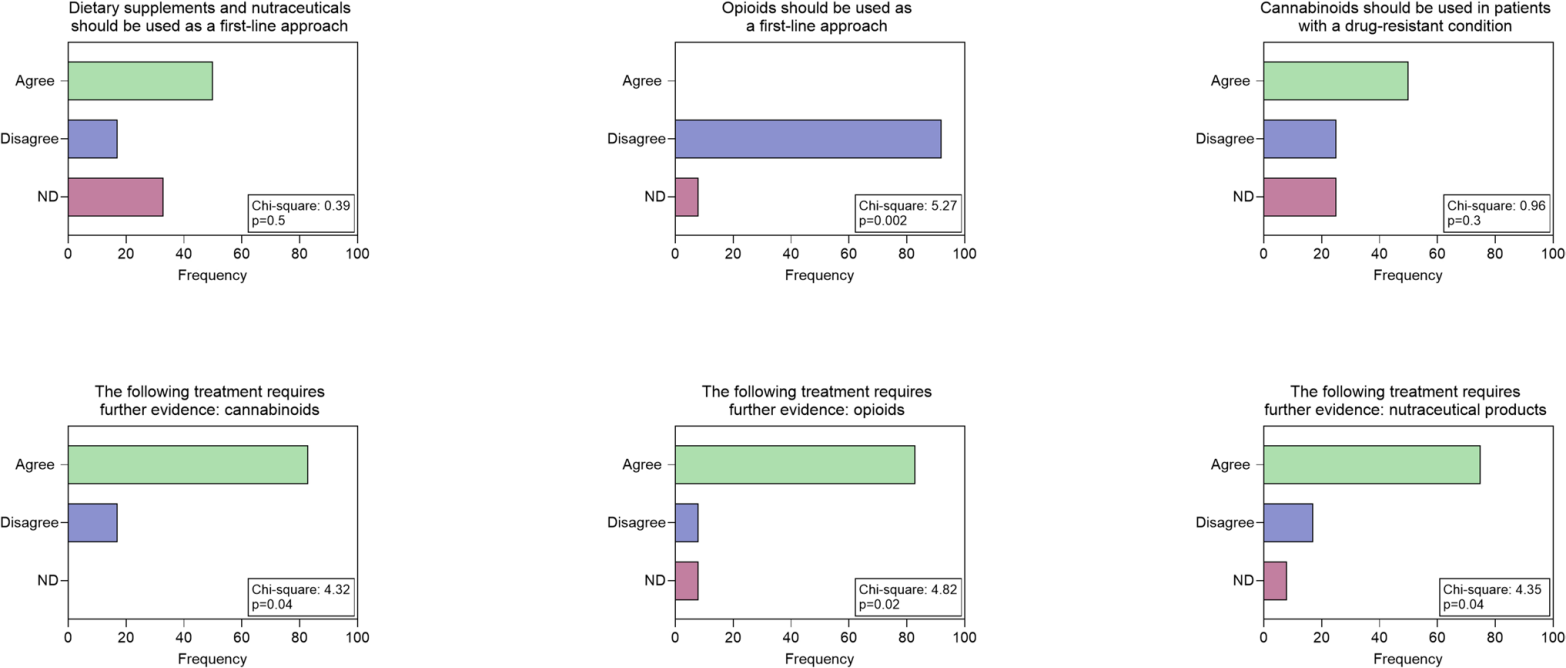
Answers to questions related to pharmacological therapies: Chi-square test results are reported

**Fig. 3:**
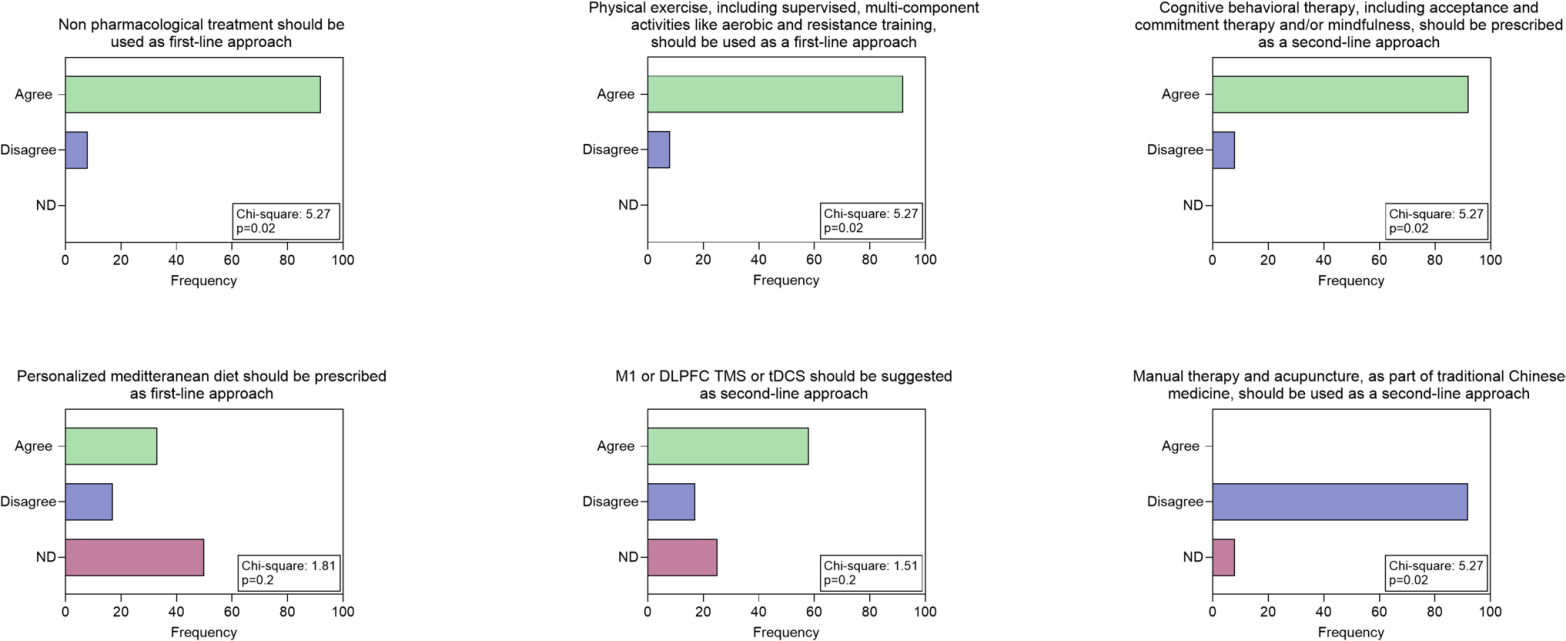
Responses to questions related to non-pharmacological treatments: Chi-square test results are reported

In terms of pharmacological treatment, there was no clear consensus on the possibility of prescribing medication in the first approach: however, the group agreed on the choice between antiepileptics or antidepressants basing on clinical judgment about comorbidities for anxiety and depression (Fig. 1). The use of opioids as first choice is discouraged unless further studies are conducted, including other mild opioids such as tramadol and tapentadol (Fig. 2). The group does not advocate the prescription of nutraceuticals as first-line treatment, but recommends further systematic studies. (Fig. 2). As a second choice, experienced neurologists recommend the association between antiepileptic drugs and antidepressants, while they disagree with cannabis use, but consider it appropriate to design further more systematic studies (Figs. 1 and 2). Regarding the non-pharmacological approach, neurologists agree to suggest it in the first visit in the form of physical activity, especially aerobic exercise and strength training (Fig. 2). For the second option, they agree on the benefits of a cognitive behavioral approach but the most of them disagreed with the indication of acupuncture. (Fig. 2). For diet and NIBS, no consensus was reached on the potential benefits of a second-choice approach (Fig. 3).

**Fig. 4:**
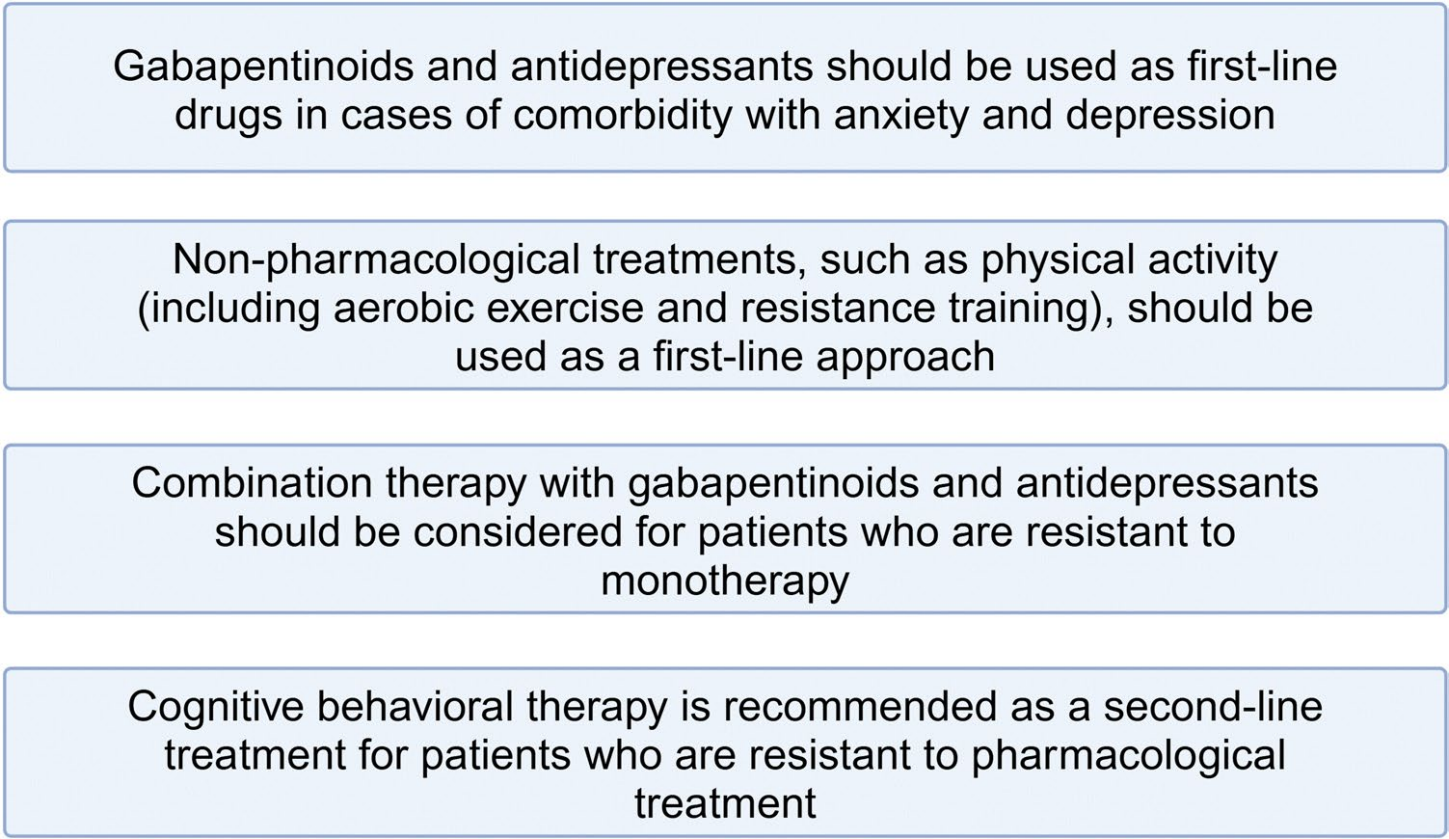
Summary of recommendations for the therapeutic approach to fibromyalgia. A)

**Fig. 5:**
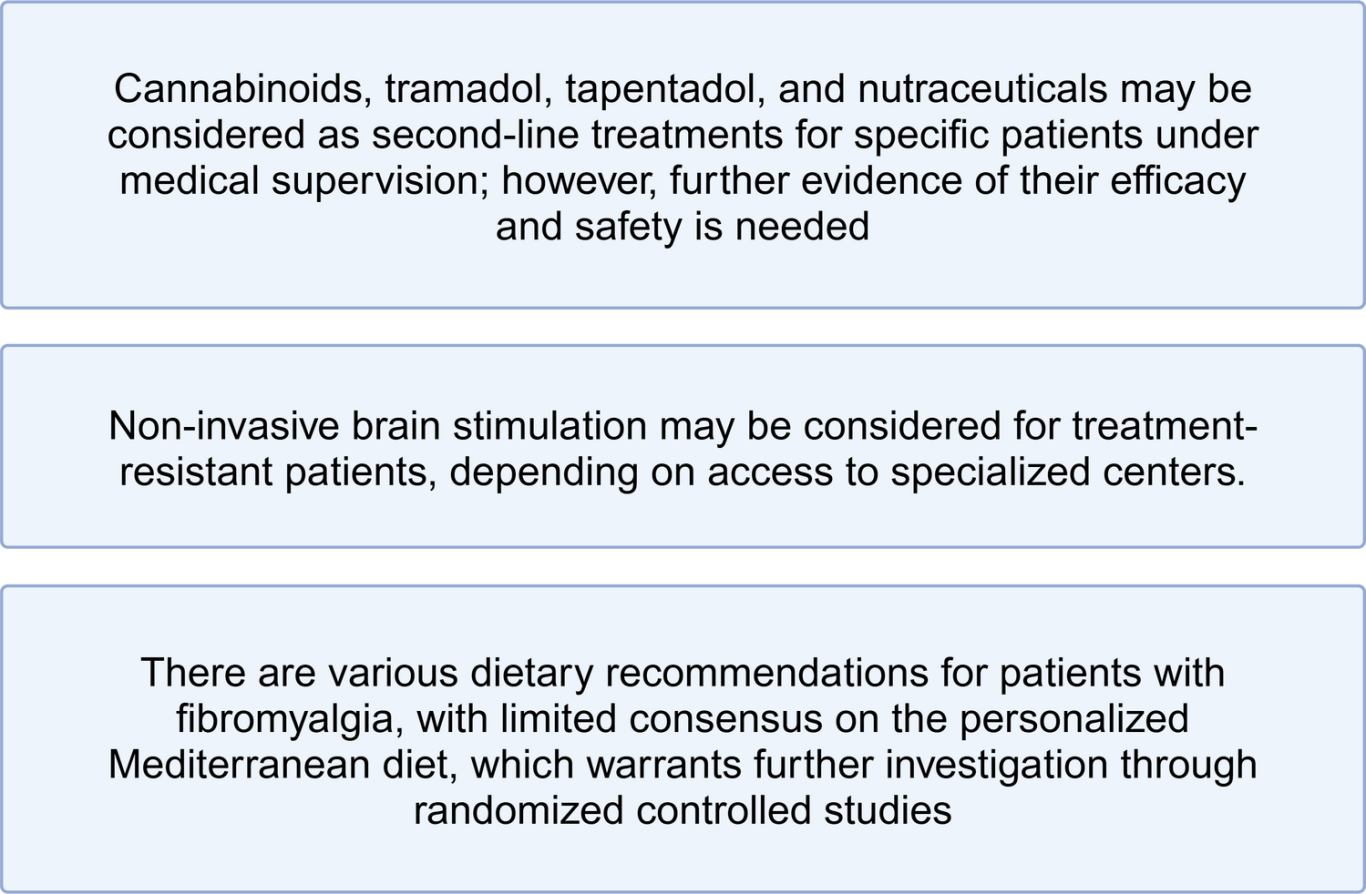
Summary of recommendations for the therapeutic approach to fibromyalgia. B)

Figures 4 and 5 show the final recommendations.

To summarize, the use of antiepileptic drugs or antidepressants is not recommended as a first choice, while their use in case of comorbidity with anxiety and depression is based on medical judgment. Association is recommended when monotherapy fails.

Non-pharmacological treatments - aerobic exercise and resistance training - are recommended as first choice.

As a second-line treatment, neurologists suggest cognitive behavioral therapy.

Medicinal cannabis, tramadol or tapentadol, nutraceutics, are worth further controlled trials and could be used in individual patients under medical supervision. (Fig.4, Fig 5)

## Discussion

With this study, the Neuropathic Pain Interest Group of the Italian Society of Neurology aimed to provide neurologists with practical guidelines for the treatment of fibromyalgia, a condition in which the involvement of the peripheral and central nervous system has been confirmed in several studies [2]. Previously, the same group had already published practical recommendations on the diagnostic procedures useful for the clinical assessment of fibromyalgia [3].

The group came to a consensus based on their clinical impression and supported by a review of the most recent randomized controlled trial, which was selected on the basis of a joint assessment of validity for clinical purposes.

The conclusions did not differ from those formulated by experienced rheumatologists [4] and previous guidelines [116]. The EULAR recommendations are based on a systematic review of the evidence since 2015, whereas we undertook a more flexible, narrative review of recent RCTs. As previously reported by other authors [117], the global approach in patients with fibromyalgia is particularly challenging, as the present study was able to confirm the lack of a specific pharmacological approach and the need for a multidisciplinary intervention, which is difficult to implement in current clinical practice. In relation to the EULAR recommendation, where no consensus was reached on the possibility of using CNS-acting medications in the treatment of comorbidities such as sleep disorders, neurologists agreed on the choice between antidepressants or anticonvulsants on medical judgment when there is a comorbidity for depression or anxiety. Given the prevalence of anxiety and depression in these patients, which ranges from 13 to 80% [118], antidepressants and antiepileptic drugs, which have been shown to be effective in the majority of patients, could also have a positive effect on pain symptoms and could be an indication for pharmacological treatment at the first approach. Furthermore, the group agreed on the efficacy of combination therapies with antiepileptics and antidepressants when monotherapy fails. Muscle relaxant drugs are commonly used in patients with FM, but the lack of studies in recent years and the lack of studies in the years before that does not even allow to formulate an opinion on their indication. In line with the rheumatologists' guidelines [4], our group has also not recommended and even discouraged the habitual use of opioids and has not reached a positive consensus on medical cannabis and nutraceuticals as first- or second-line therapies. However, they are confident about good patient compliance with medical cannabis and nutraceuticals, which is an important point in patients with FM to avoid the common nocebo effect [126]. For these reasons, the group advocates further RCTs in a global scenario of paucity of target therapies.

Similar to the recommendations of the rheumatologists [4], our group of neurologists also recommended physical activity as the first measure. Following the recommendations of physical activity experts, the group recommended a personalized treatment based on supervised aerobic exercise and strength training with an individualized combination of several components. This is a practical proposal that can be implemented with the support of sports physicians as part of an adapted physical activity program. These programs could be performed in private gyms with additional costs for the patients, but they could also be performed at home after a preliminary examination [52].

The cognitive behavioral approach is also recognized as beneficial for patients with fibromyalgia, although it requires expert support that is very rarely available in our public health system. For these reasons, although the group reached a large consensus on its effectiveness, it was proposed as a second choice. However, the current consensus suggests that the non-pharmacological approach needs to be strengthened in the public health system in general. This also applies to NIBS, which, despite being supported by several studies in terms of efficacy and safety, is not accessible in public structures.

In contrast to the non-pharmacological interventions mentioned above, acupuncture and diet were not positively evaluated as first- or second-line interventions. The reason for this could be the use of different acupuncture procedures based on the individual experience of professionals, which our group could not rely on. Different diets were also investigated and the variability of the proposed dietary approach may currently discourage the recommendation of specific dietary indications. The Mediterranean diet is the easiest to follow in our country, so only limited agreement could be reached on its applicability in the clinical management of FM patients.

Study limitations The study was based on a non-systematic review of the current literature, which served to update the general knowledge of RCTs and to formulate practical guidelines. These are not based on evidence but on the subjective agreement of neurologists specializing in neuropathic pain.

## Conclusions

There is still no specific therapeutic approach for FM based on causal mechanisms. In a general scenario of conflicting opinions about the pathophysiology and management of this complex disease, together with a general confusion about neurological expertise, the present consensus could be of help to confirm the role of the neurologist in the clinical management of patients with FM. Pharmacologic treatment with antiepileptics and antidepressants in patients with co-occurring anxiety and depression and an early non pharmacologic approach based primarily on physical exercise could be a useful indication in current neurology clinical practice. A better organized public health system, including more accessible non-pharmacological options such as cognitive behavioral therapy and NIBS, could improve evidence of efficacy and lead to relevant improvement in FM-related disability.
